# Linking genomic reorganization to tumor initiation via the giant cell cycle

**DOI:** 10.1038/oncsis.2016.75

**Published:** 2016-12-19

**Authors:** N Niu, J Zhang, N Zhang, I Mercado-Uribe, F Tao, Z Han, S Pathak, A S Multani, J Kuang, J Yao, R C Bast, A K Sood, M-C Hung, J Liu

**Affiliations:** 1 Department of Pathology, The University of Texas MD Anderson Cancer Center, Houston, TX, USA; 2 Department of Molecular and Cellular Oncology, The University of Texas MD Anderson Cancer Center, Houston, TX, USA; 3 Department of Genetics, The University of Texas MD Anderson Cancer Center, Houston, TX, USA; 4 Department of Experimental Therapeutics, The University of Texas MD Anderson Cancer Center, Houston, TX, USA; 5 Department of Gynecologic Oncology and Reproductive Medicine, The University of Texas MD Anderson Cancer Center, Houston, TX, USA; 6 Center for Molecular Medicine and Graduate Institute of Cancer Biology, China Medical University, Taichung, Taiwan

## Abstract

To investigate the mechanisms underlying our recent paradoxical finding that mitotically incapacitated and genomically unstable polyploid giant cancer cells (PGCCs) are capable of tumor initiation, we labeled ovarian cancer cells with α-tubulin fused to green fluorescent protein, histone-2B fused to red fluorescent protein and FUCCI (fluorescent ubiquitination cell cycle indicator), and tracked the spatial and time-dependent change in spindle and chromosomal dynamics of PGCCs using live-cell fluorescence time-lapse recording. We found that single-dose (500 nm) treatment with paclitaxel paradoxically initiated endoreplication to form PGCCs after massive cell death. The resulting PGCCs continued self-renewal via endoreplication and further divided by nuclear budding or fragmentation; the small daughter nuclei then acquired cytoplasm, split off from the giant mother cells and acquired competency in mitosis. FUCCI showed that PGCCs divided via truncated endoreplication cell cycle (endocycle or endomitosis). Confocal microscopy showed that PGCCs had pronounced nuclear fragmentation and lacked expression of key mitotic proteins. PGCC-derived daughter cells were capable of long-term proliferation and acquired numerous new genome/chromosome alterations demonstrated by spectral karyotyping. These data prompt us to conceptualize a giant cell cycle composed of four distinct but overlapping phases, initiation, self-renewal, termination and stability. The giant cell cycle may represent a fundamental cellular mechanism to initiate genomic reorganization to generate new tumor-initiating cells in response to chemotherapy-induced stress and contributes to disease relapse.

## Introduction

Cell cycle represents a series of events that take place in a cell to faithfully replicate the genetic materials and to distribute them to the daughter cells. Proper regulation of cell cycle represents most fundamental mechanism for normal development and prevention of neoplasia in eukaryotic organisms. The best known cell cycle is mitotic cell cycle, which involves several distinct phases including DNA synthesis (S) and distribution of replicated DNAs to two identical daughter cells via mitosis (M) with the intervening gap phase (G). However, during normal development and organogenesis, cells can go through an alternative cell cycle named endoplication cell cycle via either S/G without mitosis named endocycle or enter mitosis but fail to complete all aspects of mitosis without cell division named endomitosis. Continued DNA replication via endoreplication cell cycle invariably leads to a polyploid genome and an increase in cell size to generate mono- or multinucleated giant cells.^1, 2, 3, 4^ The endoreplication cell cycle and their variants play important role in Drosophila and plant development, several mammalian cells organs including megakaryocytes, placenta and liver.^1, 2, 3, 4, 5^

The role of polyploidy remains controversial in cancer development. The polyploid genome has been found in approximately 37% of all human tumors.^6^ Mononucleated or multinucleated polyploid giant cancer cells (PGCCs) are common in many high-grade cancers and chemoresistant cancers.^7, 8, 9, 10^ PGCCs can suppress tumor growth because they lack the ability to execute mitosis and therefore are prone to death^11, 12, 13^ and therapy-induced senescence.^14, 15^ On the other hand, tetraploid cells have been reported to facilitate cancer cell survival and promote transformation.^16, 17, 18^ Regrowth from giant cells via de-polyploidization terminated by budding of the daughter cells has been observed in senescent fibroblasts^19^ and in cancer cells after radiation therapy,^20, 21^ chemotherapy^22, 23, 24, 25, 26^ and *RAS* oncogene activation.^27^ Polyploidy can facilitate senescence-induced replication barrier and promote tumor progression.^28^ Whole-genomic doubling has been shown to accelerate cancer genomic evolution.^29^ Giant cancer cells have even been reported to contribute to metastasis.^30^ These data suggest that PGCCs can either suppress or promote tumor growth depending on specific cellular contexts. Recently, in a series of papers from our laboratory,^25, 26, 31, 32^ we showed that PGCCs are capable of tumor initiation and embryonic-like differentiation. Our findings raise an intriguing question of how genomically unstable and mitotically incompetent PGCCs are capable of performing these functions that require mitotic division.

In this work, we tracked the fate of PGCCs at the single-cell level following treatment with paclitaxel (PTX) to completely disable the mitotic spindle. Our findings revealed a multistep programmed process and results in generation of and mitotically competent tumor-initiating cells; we refer to this process as the giant cell cycle.

## Results

### PGCCs growth after PTX treatment

**Figure 1:**
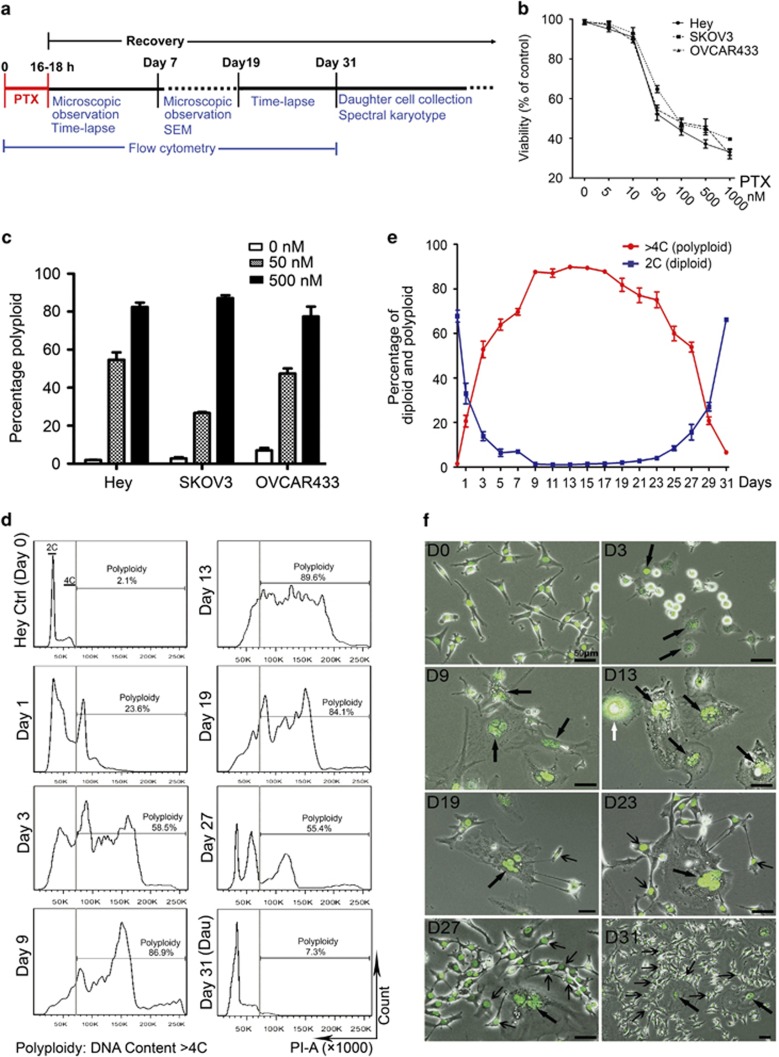
Growth of PGCCs after treatment with Paclitaxel (PTX). (**a**) Experimental design. Hey, SKOV3 and OVCAR433 ovarian cancer cells were exposed to PTX and allowed to recover in regular medium. The experiments were conducted over 31 days following treatment with PTX. Microscopic observation, flow cytometry, single-cell time-lapse recording, daughter cell collection and SKY were performed at the time points indicated. (**b**) Cell viability after treatment with PTX at the indicated concentrations and recovery for 48 h in regular medium, detected by MTS. (**c**) Percentage of polyploid cells after treatment with PTX at the indicated concentrations and recovery for 7 days, quantified by FACS. (**d**) Quantization of polyploidy by PI-FACS analysis in Hey cells after treatment with 500 nm PTX. Dau, daughter. (**e**) Percentage of polyploid cells (red curve) and 2N cells (blue curve) in Hey cells after treatment with 500 nm PTX, quantified by PI-FACS. (**f**) Morphologic change in Hey cells by conventional light microscopy after treatment with 500 nm PTX. Hey cells were labeled with H2BGFP and photographed on the days when flow cytometry was performed. Bold black arrows indicate mononuclear and multinucleated PGCCs; thin black arrows indicate daughter cells. White black arrow in panel D13 indicates a PGCC going through multipolar mitosis. D, day. Bars, 50 μm.

The experimental design is shown in Figure 1a. We treated Hey, SKOV3 and OVCAR433 ovarian cancer cells with PTX for 16–18 h (overnight) and then monitored them by flow cytometry, light microscopy, fluorescent-labeled single-cell time lapse and confocal microscopy for up to 31 days. In this paper, day 0 refers to cells before treatment; day 1 refers to the first day after exposure to PTX (recovery day 1). When cells were exposed to PTX (0–1000 nm) and allowed to recover for 48 h in regular medium, cell death increased with PTX concentration, and the concentration that led to 50% inhibition of cell viability (IC_50_) was 100 nm (Figure 1b). Next, we treated the cells with PTX at 50  and 500 nm, below and above the IC_50_. The highest increases in polyploidy were observed in cells treated at the 500 nm concentration (Figure 1c), nearly all diploid cells were dead. The serum concentration of paclitaxel in clinically treated patients ranges from 80 to 280 nm,^33^ however, the intracellular concentration in brain tumor can be as high as 3 μm after a 3-h administration of the drug (175 mg m^−^^2^).^34^ We believe that this concentration of PTX was close to that *in vivo* and decided to use 500 nm paclitaxel for future experiments.

To determine how PGCCs change over time, we measured the polyploid population (defined by DNA content ⩾4C) in Hey cells over 31 days after PTX treatment. On day 0, the percentage of diploid cells (DNA content 2C) (G1) was 67.7%, and the percentage of polyploid cells was 2.1% (Figures 1d and e). The percentage of polyploid cells was 86.9% on day 9, and remained relatively stable through day 19 (84.1%), and then started to fall and leads to increase to 2C population, such that the percentage of polyploid cells was 77.8% on day 21, 55.4% on day 27 and 7.3% on day 31 (Figures 1d and e). The change in the percentage of diploid cells over time was a mirror image of the change in the percentage of polyploid cells (Figure 1e). Compared with cells treated with PTX only, cells treated with PTX and 0.5 nm aphidicolin (an inhibitor of DNA synthesis, AC) on day 0 had a significant lower percentage in the S phase on day 2 ( mentary Figures S1A and B, *P*<0.05). However, administration of aphidicolin alone overnight had no significant effect on polyploidy percentage of test cell lines compared with the control (Supplementary Figure S1B). DNA was actively replicated in the first 3–4 days and remained active at low level (Supplementary Figure S1C). These results demonstrated that there was low but active DNA synthesis contributed to the growth of polyploid tumor cells after PTX treatment.

We correlated morphology observed on light microscopy with flow cytometry. As shown in Figure 1f, on day 0, Hey cells were mononuclear. On day 3, many cells died, but we observed enlarged cells with single or multiple nuclei (Figure 1d). By day 13, almost all remaining cells were giant cells. On days 19 and 23, we observed budding of small daughter cells from PGCCs. On day 27, the mother PGCCs were surrounded by multiple budded small daughter cells and by day 31, small daughter cells had actively divided to generate their own progeny cells. Taken together, our data suggest that cancer cells treated with PTX can enter a state of slow self-renewal and grow as PGCCs and then return to non-polyploid growth after PTX treatment. Here, we refer to this entire process as 'the giant cell cycle'.

### PGCC formation and division

**Figure 2:**
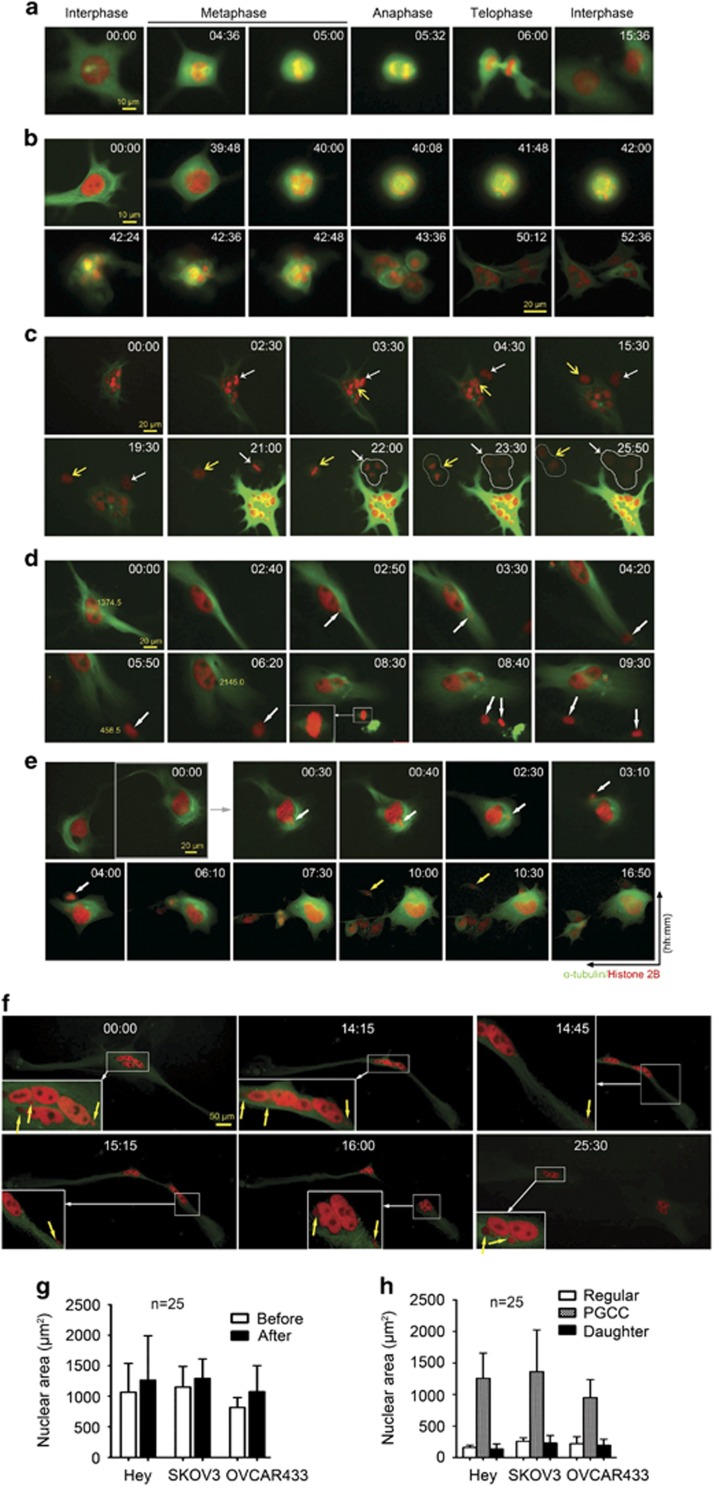
Representative single-cell time-lapse recording of PGCC formation and budding in Hey cells with DNA labeled with H2B-mCherry and tubulin labeled with Alpha-Tubulin-EGFP. (**a**) Mitotic cell cycle of regular Hey cells. Shown are chromosomes (red fluorescence) and spindle (green fluorescence) in different phases, including interphase, metaphase, anaphase and telophase. (**b**) Endoreplication followed by multipolar mitosis to generate multinucleated PGCCs. A multipolar spindle with chromosomes aligned along metaphase plates was observed at 40:00 (hh:mm), three daughter cells were visible at 43:36 and the giant cell had fully split into three daughter cells, each with multiple nuclei, at 52:36 (recorded from day 7). (**c**) Budding of two nuclei (white and yellow arrows) from a multinucleated PGCC. The budded daughter cells continued bipolar or tripolar mitotic division. White circles indicate daughter cells (recorded from day 19). (**d**) Nuclear budding (white arrows) from a mononucleated PGCC and generation of mitosis-competent daughter cells. The daughter cells resumed mitotic division as indicated by the green-dyed spindle at 08:30 (inset). White arrows also mark other nuclear changes, including focal condensation, bulge and segregation of chromosomes from the mother nucleus in the absence of a spindle. Numbers indicate total area of mother and daughter nuclei in μm^2^ calculated by Axio Vision 4.0 (recorded from day 7). (**e**) Stochastic budding of multiple nuclei (white arrows) from one of two giant nuclei of a giant cancer cell followed by sequential budding of multiple daughter cells. One of the daughter cells was a spindle-shaped cell (yellow arrow) (recorded from day 19). (**f**) A PGCC with multiple nuclei divided into two daughter PGCCs via cytofission in the absence of a spindle structure, and multiple small daughter nuclei budded off from multinucleated mother PGCC. Insets, magnified view from low magnification (white arrow); budded nuclei from mother PGCC (yellow arrows, recorded from day 19). (**g**) Nuclear area of PGCCs before and after budding, based on 25 PGCCs. (**h**) Nuclear area of regular control cells, PGCCs and daughter cells based on 25 PGCCs.

To determine the spatial and temporal dynamics of chromosome and spindle movement of PGCC, we performed single-cell time-lapse recording after labeling the chromosomes with chimeric Histone H2B-mCherry (red) and the spindle with α-tubulin-EGFP (green). Without PTX treatment, as expected, Hey cells divided via traditional mitotic cell cycle with metaphase, anaphase and telophase (Figure 2a and Supplementary Movie S1).

Next, we tracked the fate of PGCC following PTX treatment. As shown in Figure 2b (Supplementary Movie S2), a surviving cancer cell was blocked at the mitosis and switched endoreplication and result in increase in nuclear size (Figure 2b, 39 h 48 min [39:48]). Then, endoreplicated cell underwent multipolar mitosis to give rise to three multinucleated giant cells. When we tracked the fate of multinucleated PGCCs (Figure 2c and Supplementary Movies S3A and B), we found that two nuclei (white and yellow arrows) budded sequentially from a multinucleated PGCC and then underwent mitosis or tripolar mitosis to generate two or three daughter cells, respectively (white circles), while multinucleated mother cells continued to increase in tubulin and nuclear size but cell failed to separate and eventually died by apoptosis (Supplementary Movie S4)

We also tracked the fate of mononucleated PGCCs. As shown in Figure 2d (Supplementary Movie S5A), a mononucleated PGCC showed focal asymmetrical condensation of chromosomes and segregation of a small nuclear bulge (3–5 μm) from giant mother nucleus, which traveled within the cytoplasm toward the cell membrane until it completely budded off from the mother cell. The daughter cell then grew in size and underwent mitosis and separated into two new daughter cells (Figure 2d, 08:40; Supplementary Movie S5B). Remarkably, the nuclear area of the mother giant cell continued to enlarge even after budding of the daughter cell, increasing from 1374.5 μm^2^ before (00:00) to 2145.0 μm^2^ after (06:20) budding of the daughter cell (Supplementary Movies S5A and 5B), which had nuclear area of 458.5 μm^2^ (05:50). Such findings suggested that endoreplication cell cycle continued during budding of daughter cells.

We also observed similar asymmetrical budding of multiple daughter cells from a giant mother nucleus (Figure 2e and Supplementary Movies S6A and 6B), one of the daughter cells also displayed spindle morphology (Figure 2e, 10:00 and 10:30, yellow arrows). The process from asymmetrical condensation of chromosomes to complete budding off of the first daughter cell took about 4 h. Multiple buddings were then observed over the next 6 h.

We also observed split division of PGCCs. As shown in Figure 2f (Supplementary Movie S7), the PGCC split in the middle of the giant nucleus (nuclear fission) and then separated into two daughter cells (cytofission) (Figure 2f, 16:00, 4% of total PGCCs), while multiple small nuclei were budding off from a giant nucleus and continuously moving toward the cell surface.

A total of 30 movies with about 100 randomly selected PGCCs starting on day 19 for 96 h were observed and analyzed. Multipolar mitosis, budding and cytofission counted for 53.3, 6.7 and 6.3%, respectively. Analysis of the nuclear areas of 25 PGCCs from each cell line analyzed (Hey, SKOV3 and OVCAR433) before and after budding showed that the nuclear area consistently increased after budding (Figure 2g), demonstrating that PGCCs continued to grow via endoreplication cell cycle while budding off daughter cells. The daughter cells immediately after budding were consistently smaller than the control cells (Figure 2h).

### Endoreplication revealed by FUCCI

**Figure 3:**
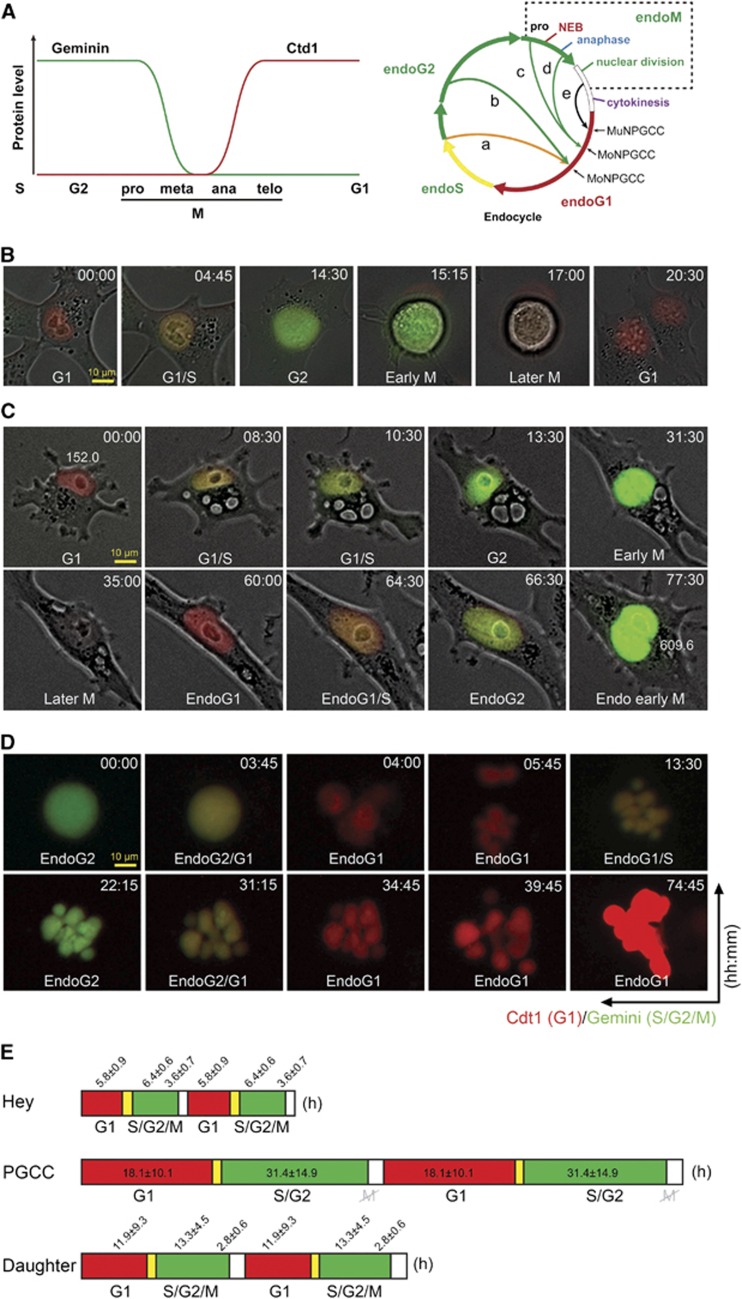
Representative endoreplication in PGCCs labeled with FUCCI. (**A**) Schematic showing how FUCCI is used to detect cell cycle phase. Left: Levels of geminin (green curve) and Cdt1 (red curve) are plotted on the vertical axis. The different phases of the mitotic cell cycle is plotted on the horizontal axis. The color of fluorescence indicates the cell cycle phase (G1, red; S, orange/yellow; G2/early M, green; late M, colorless). Right: The endoreplication cell cycle can be truncated at any of several different phases after S. Different endoreplication cell cycles are indicated by the arrows labeled a to e. The endocycle involves oscillations between a G phase and S phase either endoS/G1 (a) or endoS/G2/G1 (b) without entering mitosis and will generate mononucleated polyploid or polytene giant cancer cells (MoNPGCC). c to e, endomitosis. The definition is broadened to include entry into mitosis but failure in all aspects of mitosis as recently described.^3^ This can involve failure of nuclear envelope breakdown but assembly of a spindle within the nucleus and segregation of sister chromatids, nuclear envelope breakdown, anaphase and/or nuclear division but not followed by cytokinesis (a: cytoplasmic mitosis). c: endoS/G2/M/G1 without nuclear membrane breakdown (NEB); d, endoS/G2/M/G1 with NEB; c and d will produce mononucleated PGCC (MoNPGCC); e. EndoS/G2/M/G1 cycle will generate multinucleated PGCC (MuNPGCC) following nuclear division. (**B**) Mitotic cell cycle indicated by FUCCI. G1, red; G1/S, yellow; G2, green; early M, green, ball- shaped; late M, ball-shaped, colorless; and G1, red. The typical mitotic cell cycle is G1/S/G2/M. (**C**) Mononucleated Hey cell before and after two rounds of endoreplication without cell division. Numbers in the first and last panels indicate total nuclear area in μm^2^ calculated using Axio Vision 4.0 (Zeiss, Thornwood, NY, USA). The recording PGCCs in **C** and **D** started at day 1 following paclitaxel treatment. (**D**) Mononucleated PGCC divided via endomitosis to generate multinucleated daughter cells that continued endocycle. (**E**) Pattern and lengths (mean±s.d.) of different phases of mitotic cell cycle of regular Hey and daughter cells and giant cell cycle of PGCCs, based on analysis of 25 cells.

To further clarify the mode of PGCCs division, we labeled Hey cells with fluorescent ubiquitination cell cycle indicator (FUCCI).^35^ FUCCI employs red fluorescent protein fused to the cell cycle indicator Cdt1 and green fluorescent protein fused to the cell cycle indicator geminin to indicate cell cycle phase (Figure 3A). Cells in G1 are red, cells in the G1 to S transition are yellow, cells in S, G2 and early M are green, and cells in late M to early G1 are colorless (Figure 3B and Supplementary Movie S8).

As shown in Figure 3C (Supplementary Movie S9), a mononuclear Hey cell exhibited two changes from red to green (two rounds of DNA replication) in the absence of mitosis (20% of PGCCs), resulting in a quadrupling of the nuclear area, from 152.0 to 609.6 μm^2^.

Also, as shown in Figure 3D, a mononucleated PGCC at endoG2 generated multinucleated PGCCs followed budding (Supplementary Movie S10) that continued endocycle (40% of PGCCs), confirming that Hey cells surviving after PTX treatment underwent endoreplication. Additional examples of nuclear fragmentation and endoreplication are shown in Supplementary Movies S11 and S12. The example of PGCC-derived daughter cells resumed mitotic division is shown in Supplementary Movie S13.

Quantitative analysis of cell cycle length based on data from time-lapse recording in 25 Hey PGCCs and daughter cells that completed the entire endoreplication cell cycle is summarized in Figure 3E. The duration (mean±s.d.) of the entire mitotic cell cycle was 17.9±4.3 h. The lengths (mean±s.d.) of the various cell cycles were as follows: endoreplication cell cycle of PGCCs, 49.5±24.9 h; mitotic cell cycle of regular Hey cells, 17.9±4.3 h; and mitotic cell cycle of Hey daughter cells, 28.1±9.5 h. There were enormous variations in different phases of cycle length within the giant cell cycle as compared with that of the mitotic cell cycle (Figure 3E).

### Shut down of mitotic division in PGCCs

**Figure 4:**
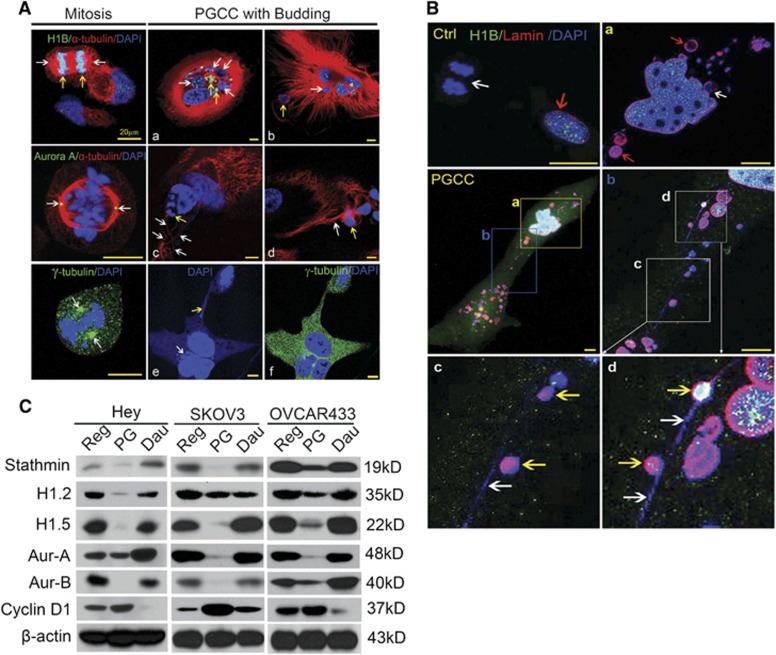
Confocal microscopic analysis of mitotic regulatory proteins in PGCCs. (**A**) Confocal images of PGCCs undergoing budding in Hey cells. (Left upper image) White arrows indicate the spindle (α-tubulin); yellow arrows indicate the anaphase chromosomes (H1B) collected on the metaphase plate. (Left middle and lower images) Aurora A expression in the spindle pole (middle image) and γ-tubulin expression in centrosomes (lower image); white arrows indicate the centrosomes to which the mitotic spindle attached. (a and b) Multiple variable-sized nuclei encased within a microtubular nest (a, white arrows) with H1B expression (yellow arrow). A near-budding nucleus (b, white arrows) wrapped by tubulin (b, yellow arrow) indicated discontinuous tubulin surrounding the daughter nuclei. (c) Multiple budding nuclei (yellow arrow) broken off from a giant mother nucleus. White arrows indicate the interlaced channel on the longitudinal section through which nuclei were transported. (d) Thin-thread-like tubulin bridge (white arrow) connecting a daughter cell (yellow arrow) to a multinucleated nucleus. (e) Multiple fragmented nuclei (white arrow) connected through a thin-thread chromatin bridge (yellow arrow). (f) Overlaying of γ-tubulin with DNA staining indicates lack of centrosome-like structure. Bars, 20 μm. (**B**) Nuclear membrane structure in regular Hey cells (Ctrl) and a PGCC with budding (a–d). (Ctrl) Absence of a nuclear membrane in the mitotic nucleus (white arrow) and presence of a nuclear membrane in the interphase nucleus (red arrow) in regular Hey cells. (PGCC) PGCC with budding at a low magnification. The background of green H1B was adjusted to show the outline of a single PGCC. Regions a and b were magnified to show the details of chromosomal bridge composed of thread-like chromosomes and budding nuclei. (a) Inset from PGCC, region a. Fragmented nuclei within the PGCC. Red arrows, daughter nuclei with intact nuclear membrane; white arrow, semi-attached daughter nucleus with partial nuclear membrane. (b) Inset from PGCC, region b. The contour of the nuclear membrane is highlighted with anti-lamin monoclonal antibody. (c and d) Insets from panel b. Thin-thread chromatin links budding nuclei. DNAs present as thin-thread DNAs (white arrows in c and d) within cytoplasmic branch of daughter cells. Yellow arrow, daughter nucleus budded out from thin-thread DNAs covered with intact nuclear membrane. Bars, 20 μm. (**C**) Protein expression of cell cycle and mitotic molecules in test cells detected by western blotting. Aur-A, Aurora A; Aur-B, Aurora B; Dau, daughter cells budded off from PGCC at recovering day 28; H1.2, Histone 1.2; H1.5, Histone 1.5; PG, PGCCs at recovering day 7 following paclitaxel treatment; Reg, regular cancer cells.

To determine the molecular mechanisms involved in formation of PGCCs, we used confocal scanning microscopy following green immunofluorescence labeling of Hey cells with Histone 1B (H1B), Aurora A kinase and γ-tubulin. During mitosis, H1B was highly expressed and co-localized with chromosomes in the middle plate (Figure 4A, left upper image, yellow arrow), Aurora A was detected at the spindle pole in centrosomes (Figure 4A, left middle image, white arrow) and assembly of centrosome γ-tubulin was readily visible on both sides of the aligned chromosomes (Figure 4A, left lower image, white arrow). In contrast, in PGCCs, no spindle-like structure was detected; instead, α-tubulin formed a microtubular cage surrounding giant nuclei together with budded nuclei (Figure 4Aa, white arrows). There were only scattered heterogeneously H1B-positive granules scattered within some nuclei (Figure 4Aa, yellow arrows), suggesting that chromatins are not packed with histone. In addition, there was no Aurora A expression (Figures 4Aa and d). The budded cells were surrounded by a microtubular mesh composed of discontinuous α-tubulin as indicated by red fluorescence on the horizontal section and microtubular braid on the longitudinal section (Figure 4Ac, white arrows) and linked by a thin fibrous bridge (Figure 4Ad, white arrow). In addition, the nucleus was also fragmented into variably sized daughter nuclei (Figure 4Ae, white arrow), which are connected to a thin chromatin bridge (Figure 4Ae, yellow arrow). The process did not involve assembly of centrosomes, as indicated by the lack of γ-tubulin staining (Figure 4Af).

Examination of nuclear membrane status showed that regular Hey cells (Figure 4B, panel labeled 'Ctrl') has intact nuclear membrane (red arrow) but lacked it (white arrow) during mitosis. As shown in the right middle image, numerous fragmented daughter nuclei were observed within a single PGCC, the nuclear membrane was detected in both giant nucleus and in daughter nuclei, which were further highlighted in Figures 4Ba and b. On high magnification of Figure 4Ba indicated by Figures 4Bc and d, we observed the thin chromosomal bridge (white arrows) connecting daughter nuclei (yellow arrows). These findings indicated that formation of PGCCs and the budding was not associated with breakdown of the nuclear membrane, which is absolutely required for cells to undergo mitotic cell division.

Western blot analysis showed that expression of mitosis-related proteins, including stathmin, Histone 1.2, Histone 1.5, Aurora A, and Aurora B, was consistently downregulated in PGCCs and re-expressed in daughter cells (Figure 4C). Cyclin D1, a molecule involved in cell cycle initiation, was upregulated significantly in PGCCs and downregulated significantly in daughter cells in all three tested cell lines, demonstrating that the mitotic machinery is turned off in PGCCs and reactivated after daughter cells after they were budded off from giant mother cells.

### Acquisition of new cancer genome in PGCCs-derived daughter cells

**Figure 5:**
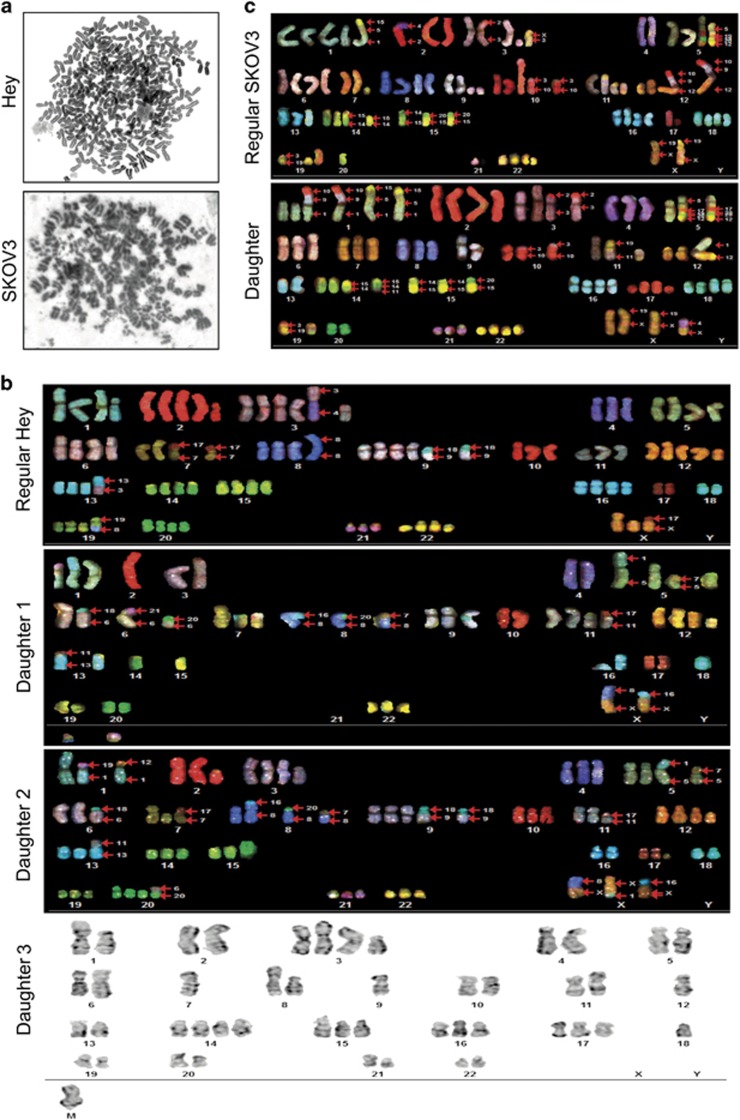
SKY analysis of regular cancer cells and PGCCs. (**a**) Endomitotic polyploid metaphases were found in Hey cells (upper) and SKOV3 cells (lower). (**b**) Difference in chromosome number between regular Hey cells and three representative daughter cells. (**c**) Chromosome number of regular SKOV3 and daughter cells.

To examine if PGCC-derived daughter cells may have acquired new genomic alterations, we performed spectral karyotyping (SKY) on PGCC and daughter cells. As shown Figure 5a, multiple endomitotic polyploid metaphases were found in PGCC from Hey and SKOV3 cells. Representative pictures of SKY analysis and daughter cells in Hey are shown in Figure 5b. We found that new multiple chromosomal rearrangements, including deletions and translocations, occurred in daughter cells as compared with parental cancer cells. In daughter cancer cells, of 111 metaphases analyzed, 100 (90%) had lower chromosome number than parental cells, with chromosome number ranging from 55 to 75, and 11 (10%) had nearly diploid chromosomes, with chromosome number ranging from 35 to 51; four cells (4%) had about 48 chromosomes (Figure 5b). Detailed SKY analysis showed that parental Hey and daughter cells shared only five chromosomal rearrangements: del(1), del(3), t(7;17), t(9;18), and del(12). In addition to these common markers, Hey parental cells had 10 chromosomal rearrangements, and Hey daughter cells had 29 (Supplementary Table S1). SKY analysis showed that parental SKOV3 cells had two chromosomal rearrangements, t(2;4) and t(15;14;16), SKOV3 daughter cells also had two rearrangements, t(4;X) and t(15;14;11) (Supplementary Table S1) while parental and daughter cells shared 11 chromosomal rearrangements (Figure 5c). The data demonstrated that daughter cells acquired a new karyotype with numerous genomic alterations following single giant cell cycle.

## Discussion

**Figure 6:**
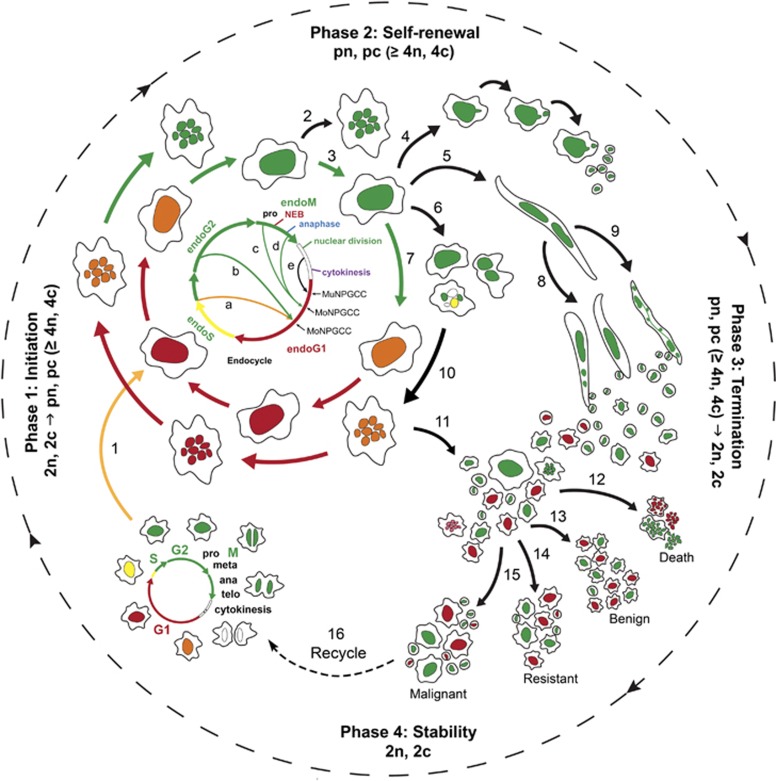
Schematic of the giant cell cycle. The mitotic cell cycle is shown in the lower left corner. In response to acute stress, massive cell death occurs, but a subset of cancer cells enters the giant cell cycle with the four phases labeled in the figure: initiation from nearly diploid (2n, 2c) to tetraploid and then polyploid (⩾4n, 4c; pn, pc), self-renewal of polyploid growth (pc, pn), termination from polyploid or tetraploid to diploid (pc, pn; 4n; 4c to 2n, 2c) and stability phase with proliferation of tumor cells via mitosis (2n, 2c). The color of fluorescence indicates the cell cycle phase (G1, red; S, orange/yellow; G2/early M, green; late M, colorless) as defined in the FUCCI experiments in Figure 3. The arrow labeled 1 indicates initiation of the endoreplication from diploid cells (2n, 2c) to become tetraploid (4n, 4c; pc, pn) after the mitosis is shut down. The other labeled arrows indicate possible outcomes after initiation of the endoreplication, as follows: 2, endomitosis to generate a multinucleated PGCC. 3, 7, and 10, continued endoreplication. 4, budding of daughter cells. 5, nuclear fission within a PGCC. 6, multipolar mitosis followed by cytofission. 8 and 9, continued fragmentation and budding of PGCC. 11, resumption of mitosis of daughter cells. 12, death from PGCC. 13, differentiation into benign stromal cells. 14, new cancer cells with newly acquired with genetic/epigenetic landscape. 15, malignant clones with potential to metastasize. 16, initiation of the giant cell cycle if cancer cells face new catastrophic event. 2n, 2c: diploid or pseudo-diploid tumor cells at the G1 phase, pn, polyploid cancer cells defined by ploidy ⩾4n, 4c at the G1 phase.

In this study, using live-cell time-lapse recording to track the dynamics of chromosomes, spindle and cell cycle, we revealed for what we believe is a detailed multistep reprogramming process by which to generate new mitotically competent tumor-initiating cancer cells, we refer it as the giant cell cycle. The giant cell cycle, a schematic of which is presented in Figure 6, can be divided into four phases, initiation, self-renewal, termination and stability.

During the initiation phase, the nearly diploid G1 cells (2n, 2c) are under attack from a life-threatening stressor, and the diploid G2 cells (2n, 4c) undergo mitotic catastrophe with massive cell death. In a subset of cells, replication is uncoupled from mitosis and cytokinesis, traditional G1/S or G2/M checkpoints are disabled and cell divisions are coupled with the suppression of the apoptosis/senescence program via downregulation of key mitotic proteins H1, Aurora A kinase and Aurora B kinase. These surviving cells are reset into endoreplication to start polyploid growth (pn, pc⩾4n, 4c).

During the self-renewal phase, almost all non-polyploid cells die. The tetraploid cells (4n, 4c) continue the endoreplication cell cycle via endocycle or endomitosis to generate mononucleated or multinucleated PGCCs and followed by cytofission to allow the cancer cells to grow in a polyploidy state.

During the termination phase, nuclei of polyploid giant nuclei (pn, pc⩾4n, 4c) undergo depolyploidization to generate diploid cells (2n, 2c). The giant nucleus gives rise to smaller nuclei via (1) nuclear budding, (2) nuclear fragmentation (burst-like division) or (3) nuclear fission followed by cytofission.

During the stability phase, diploid daughter cells (2n, 2c) have acquired a new genome (different karyotype/mutation/genomic reorganization); these cells acquire competency in mitosis and achieve stable karyotype. Continued mitotic division of newly generated tumor-initiating cells leads to increase in tumor size with predominantly near diploid cancer cells.

Definition of the giant cell cycle has several important implications regarding our understanding of drug resistance and disease relapse:

First, the giant cell cycle provides a new cellular mechanism for genomic reorganization and genomic instability. The giant cell cycle offers an efficient and rapid microecosystem to generate aneuploidy/polyploidy and facilitates fast genomic reorganization (chromosomes or genes, chromothripsis) due to DNA replication without mitotic checkpoints.^36, 37, 38^ Massive genomic rearrangement in response to stress was long ago described by McClintock.^39^

Second, the giant cell cycle serves as a novel source for mitotically competent tumor-initiating cells. PGCCs grow asynchronously in the presence of a growth-suppressive danger and are resistant to apoptosis/senescence and become more resilient to chemotherapy and other stresses. The giant cell cycle thus resolves our paradoxical finding that PGGCs are capable of tumor initiation.

Third, the giant cell cycle provides a rational explanation for why PGCCs acquire embryonic-like stemness.^26^ Depending on the stress level and type of stress, endoreplication can make multiple copies of naive new genomic DNAs and may lead to dedifferentiation into the embryonic-like cancer stem cells.

Our studies validate early findings of polyploid giant cells by other investigators and also our own laboratory. Erenpreisa *et al.*^20, 40^ observed budding from irradiated cancer cells; Walen^41, 42^ showed that giant cells can provide an alternative source for non-mitotic genomic stability. Sundaram *et al.*^24^ and Rajaraman *et al.*^43^ proposed the neosis hypothesis for giant cell division and budding and generation of transient stemness for carcinogenesis, a hypothesis that was extended by Erenpreisa *et al.*,^44, 45, 46^ who posited an evolutionarily conserved life cycle for tumor initiation. While this work is under review, Chen *et al.*^47^ reported that mitotic inhibitors can induce transient endoreplication, several rounds of division, ultimately sprawling of proliferative cells of reduced ploidy with novel genomic instability. Taken together, these data from different investigators and our own provide independent validation on the important role of PGCCs in tumor development.

In summary, we have provided strong evidence for the existence of a giant cell cycle that may constitute a generalized mechanism for survival and generation of genomically altered tumor-initiating cells that contribute to the disease relapse. Defining the molecular mechanisms that regulate the giant cell cycle should offer novel opportunities for therapeutic intervention for this devastating disease.

## Materials and methods

### Cell lines and tissue culture

Three human ovarian cancer cell lines, Hey, SKOV3 and OVCAR433, were purchased from American Type Culture Collection (Manassas, VA, USA). Hey and OVCAR433 cells were cultured in MEM Eagle (Lonza, Walkersville, MD, USA) supplemented with 10% fetal bovine serum (Gibco/Invitrogen, Grand Island, NY, USA). SKOV3 cells were cultured in RPMI-1640 plus 10% fetal bovine serum. At 60–70% confluence, cells were treated with PTX (0–1000 nm; Sigma, St Louis, MO, USA) overnight (16–18 h); cells were then allowed to recover for up to 31 days in regular medium. The origin and purity of all three cell lines were verified with short tandem repeat (STR) sequence analysis before their use in Anderson's Characterized Cell Line Core (https://www.mdanderson.org/education-and-research/resources-for-professionals/scientific-resources/core-facilities-and-services/characterized-cell-line-core-facility/index.html).

### MTS assay

To determine the sensitivity and the 50% concentration of inhibition (IC_50_) of test cell lines to different chemotherapy drugs including PTX, test cells (control Hey, Hey daughter, control SKOV3, SKOV3 daughter; 5000/well) were treated in triplicate with the PTX of increased concentrations (0, 5, 10, 50, 100, 500 and 1000 nm) overnight (16–18 h); cell viability was tested by MTS (3-(4,5-dimethylthiazol-2-yl)-5-(3-carboxymethoxyphenyl)-2-(4-sulfophenyl)-2H-tetrazolium) assay with CellTiter96 Aqueous One Solution cell proliferation assay (Promega, Madison, WI, USA) according to the manufacturer's instructions. IC_50_ values were calculated for each cell for each drug.

### Flow cytometry

Hey, SKOV3 and OVCAR433 cells were exposed to PTX at 0, 50 and 500 nm overnight, allowed to recover for 7 days in regular medium, and then collected and stained with propidium iodide (Sigma). Hey cells were exposed to 500 nm PTX overnight and allowed to recover in regular medium; cells were stained with propidium iodide every 2 days from day 1 to day 31.

To study DNA duplication, test cells were treated overnight with 500 nm PTX, PTX and aphidicolin (AC, an inhibitor of DNA synthesis, 0.5 nm, 1 h), or AC (0.5 nm, 1 h) only, and then allowed to recover in regular medium, and incubated with BrdU (30 nm; BD Biosciences, San Jose, CA, USA) for 3 h a day on days 0–7. Each day, cells were collected and examined with a FITC BrdU flow kit (BD) with flow cytometry according to the manufacturer's protocol.

### Cell labeling for cell division and time-lapse recording

To visualize PGCC division, Hey and SKOV3 cells were double-labeled with pmEGFP-alpha-tubulin-C1 (α-tubulin-EGFP) and PH2B-mCherry-IROS-puro (H2B-mCherry; Addgene), or single-labeled with pBabe-H2BGFP (Addgene) with fuGENE 6 transfection reagent (Promega) and selected with puromycin and G418 (Sigma). OVCAR433 cells were only labeled with pBabe-H2BGFP. Cells used to record cell cycle phase were infected with FUCCI 12 (Invitrogen) followed by selection and subcloning. FUCCI employs red fluorescent protein fused to the cell cycle indicator Cdt1 and GFP fused to the cell cycle indicator geminin to indicate cell cycle phase.

One hundred selected randomly PGCCs from Hey from 30 movies following PTX treatment were analyzed for different modes of division (including multipolar mitosis, budding and cytofission) and cell cycle change together with 25 regular cancer cells (control) using time-lapse recording with an AxioVision 4 microscope (Zeiss, Thornwood, NY, USA). For cell cycle change, the recording was performed immediately after treatment (recovery day 1, with an interval of 12 or 15 min), while cell division was recorded starting day 7 or day 19 (15 or 30 min). The percentage of different division mode was calculated based on total 100 PGCCs in Hey analyzed. The nuclear area of PGCCs before and after budding, the nuclear area of daughter cells and the regular control cancer cells were measured with Axio Vision 4 software based on 25 randomly selected test subgroups.

### Immunofluorescence, confocal scanning and H&E staining

To study the dividing of PGCC, immunofluorescence and confocal scanning were performed on PGCC with budding. Primary antibodies to several mitotic molecules (α-Tubulin, γ-Tubulin, H1B, Aurora A and Lamin) are listed in Supplementary Table S2. Alexa Fluor 488 goat anti-rabbit IgG (H+L) and Alexa Fluor 594 goat anti-mouse IgG (H+L) (Invitrogen) were used as the secondary antibodies. After mounting with Vectashield medium containing DAPI (Vector, Burlingame, CA, USA), slides were evaluated with a BX72 microscope (Olympus, Tokyo, Japan) or a Carl Zeiss 710 confocal microscope (Zeiss).

### PGCC and daughter cell collection and SKY

Following recovery in regular medium for 14 days, 20 individual PGCCs were picked out separately, reseeded in 96-well plates (one PGCC per well) and cultured with filtered (0.2 μm) conditioned medium for an additional 2 weeks. Single-cell selection and culturing with filtered conditioned medium guaranteed the purity of daughter cells derived from PGCCs. When the daughter cells budded out and formed clone-like populations, daughter cells from this single PGCC were dissociated and were cultured in 12-well plates to grow into stable daughter cell population. Finally, three stable daughter clones of test cells from single giant cell cycle were cultured for ⩾30 generations. Their origin and purity of parental and daughter cells were confirmed with short tandem repeat sequence analysis.

Then SKY analysis was performed on 111 randomly selected daughter cells derived from Hey and SKOV3 PGCCs and 20 regular control cells using human paint probes from Applied Spectral Imaging as per the manufacturer's recommendation. Images were captured with a Nikon 80i microscope and analyzed with SKY software (Applied Spectral Imaging). A total of 15–20 metaphases from each sample were analyzed in detail.

### Western blotting

Western blot analyses were carried out to detect the expression of molecules related to mitosis (Stathmin, Histones 1.2, Histone 1.5, Aurora A and Aurora B) and cell cycle (Cyclin D1) in test groups as described previously.^48^ Details of the primary antibodies are listed in Supplementary Table S2.

### Statistics

Nuclear area of 25 randomly selected PGCCs, PGCC-derived daughter cells, regular cancer cells of the tested cell lines, nuclear area of PGCCs and budded daughter cells, were analyzed with *t-*test or one-way ANOVA. Statistical analysis was performed. Two-sided at a 5% level of significance using SPSS software (SPSS for Windows version 22.0; SPSS Inc., Chicago, IL, USA). All quantitative results were presented as mean±s.d.

## Supplementary Material

Supplementary Movie S2

Supplementary Movie S3A

Supplementary Movie S3B

Supplementary Movie S4

Supplementary Movie S5A

Supplementary Movie S5B

Supplementary Movie S6A

Supplementary Movie S6B
