# Supplementary material for: Linking genomic reorganization to tumor initiation via the giant cell cycle
Source: Oncogenesis. 2016 Dec 19;5(12):e281–. doi: 10.1038/oncsis.2016.75 (PMC5177773; doi:10.1038/oncsis.2016.75)
Supplement: Supplementary Figure S1 [file oncsis201675x1.pdf]

Fig.S1

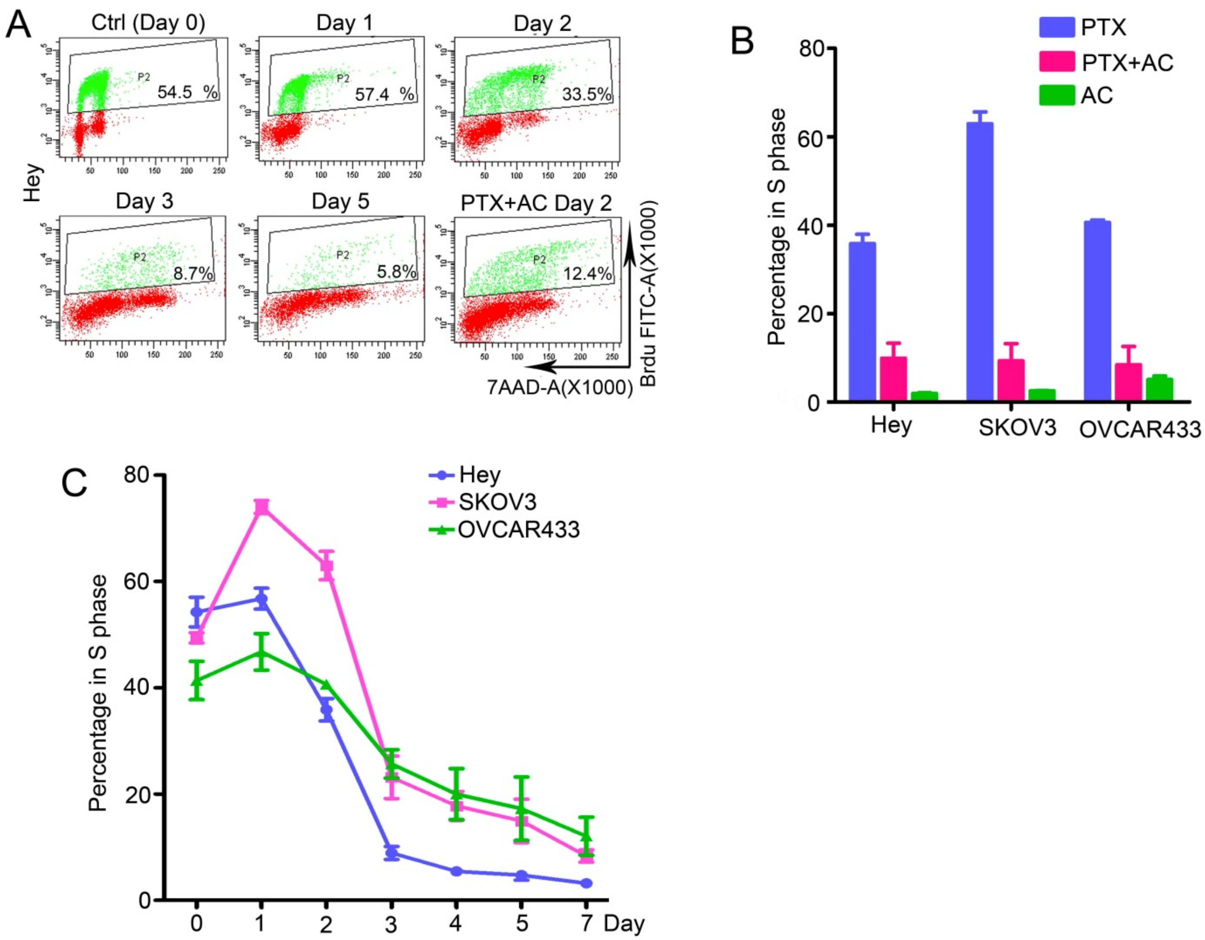

Fig. S1. Endoreplicating cancer cells have active DNA synthesis following PTX treatment.

(A) DNA replication analysis by BrdU incorporation and FACS quantization in Hey cells after treatment with 500 nM PTX. Aphidicolin (AC) inhibited DNA replication, as indicated by the results for Hey cells on recovery day 2. Green dots were gated as cell events in S phase, with regular cancer cells as a control.

(B) S phase percentage in test subgroups at recovering day 2. S phase percentage in PTX+AC is greater than that in AC only, suggesting that PTX stimulates DNA synthesis as compared with that of AC only control.

(C) Change in S phase percentage in Hey, SKOV3, and OVCAR433 cells over recovery days 0-7 after treatment with 500 nM PTX.
