# Supplementary material for: Linking genomic reorganization to tumor initiation via the giant cell cycle
Source: Oncogenesis. 2016 Dec 19;5(12):e281–. doi: 10.1038/oncsis.2016.75 (PMC5177773; doi:10.1038/oncsis.2016.75)
Supplement: Supplementary Table S1 and S2 [file oncsis201675x2.doc]

| **Table S1.** Chromosomal markers observed by spectral karyotyping analysis in Hey and SKOV3 regular and daughter cells | | | | | |
| --- | --- | --- | --- | --- | --- |
| **Common markers** | | **Unique markers** | | | |
| **Hey** | **SKOV3** | **Hey regular** | **Hey daughter** | **SKOV3 regular** | **SKOV3 daughter** |
| del(1) | t(15;5;1) | t(3;4) | t(1;19) | t(2;4) | t(4;X) |
| del(3) | t(2;3) | t(3;13) | t(1;X) | t(15;14;16) | t(15;14;11) |
| t(7;17) | t(5;17;19;20;12) | t(8;8) | t(1;13) |  |  |
| t(9;18) | t(3;10) | del(9) | t(1;21) |  |  |
| del(12) | t(11;19) | t(9;17) | t(1;5) |  |  |
|  | t(10;9;12) | t(10;15) | t(2;7) |  |  |
|  | t(15;14) | t(19;8) | del(2) |  |  |
|  | t(14;15) | t(22;17) | t(3;17) |  |  |
|  | t(15;20) | t(X;9) | t(4;7) |  |  |
|  | t(3;19) | t(X;17) | t(4;10) |  |  |
|  | t(X;19) |  | del(5) |  |  |
|  |  |  | t(5;11) |  |  |
|  |  |  | t(5;15) |  |  |
|  |  |  | t(5;7) |  |  |
|  |  |  | t(6;20) |  |  |
|  |  |  | t(6;18) |  |  |
|  |  |  | t(6;8) |  |  |
|  |  |  | del(7) |  |  |
|  |  |  | t(7;8) |  |  |
|  |  |  | t(8;16) |  |  |
|  |  |  | t(8;20) |  |  |
|  |  |  | t(8;X) |  |  |
|  |  |  | t(8;18) |  |  |
|  |  |  | t(11;17) |  |  |
|  |  |  | del(11) |  |  |
|  |  |  | der(11) |  |  |
|  |  |  | t(13;13) |  |  |
|  |  |  | del(17) |  |  |
|  |  |  | t(X; 16) |  |  |

**Table S2.** Details of primary antibodies*

| **Antibody** | **Source** | **Dilution** | **Supplier** |
| --- | --- | --- | --- |
| α-tubulin | Mouse | 1:500(IF) | Millipore, Bilerica, MA, USA |
| β-actin | Mouse | 1:5000(WB) | Sigma, St. Louis, MO, USA |
| γ-tubulin | Mouse | 1:500(IF) | Sigma |
| Aurora A | Rabbit | 1:50 (IF) | Cell Signaling |
| Aurora A | rabbit | 1:1000(WB) | Cell signaling |
| Aurora B/AIM1 | rabbit | 1:1000(WB) | Cell Signaling |
| Cyclin D1 | mouse | 1:1000(WB) | Santa Cruz, Dallas, TX, USA |
| Histone 1B | Rabbit | 1:200 (IF) | Abcam |
| H1.2 | Rabbit | 1:2000 (WB) | Abcam |
| H1.5 | Rabbit | 1:3000 (WB) | Abcam |
| Lamin A/C | Rabbit | 1:3000 (IF) | Abcam |
| Stathmin | mouse | 1:1000(WB) | Novus |

* Incubation: 4℃, overnight.

IF, immunofluresence; WB, western blot.
