# Supplementary material for: Linking genomic reorganization to tumor initiation via the giant cell cycle
Source: Oncogenesis. 2016 Dec 19;5(12):e281–. doi: 10.1038/oncsis.2016.75 (PMC5177773; doi:10.1038/oncsis.2016.75)
Supplement: Supplementary Legends [file oncsis201675x19.docx]

**Supplementary Video Legends**

**Movies S1-S7.** Hey ovarian cancer cells labeled with H2B-RFP for nucleus and α-tubulin-GFP for spindle. Movie S1, no paclitaxel treatment; Movies S2–S7, paclitaxel-treated cells.

**Movie S1.** Mitosis without paclitaxel treatment. Hey cells passed through interphase, prophase, prometaphase, metaphase, anaphase, and telophase. The chromosomes reached the maximal condensation and congressed along the spindle during metaphase, sister chromatids moved to the opposite poles in anaphase and nuclear membranes re-formed around two daughter chromosomes, and cytokinesis occurred in telophase. Total time: 23 hours: time interval: 12 min.

**Movie S2.** Formation of a single mononucleated PGCC as indicated by increase in nuclear size followed by multipolar mitosis. Total time: 53.5 hours; time interval: 12 min.

**Movie S3A.** Budding of two single nuclei from a multinucleated PGCC to form two daughter cells, which underwent bipolar or tripolar mitosis, respectively. The remaining nuclei form a rosette-like structure in mother giant cells continue to endocycle with increased accumulation of α-tubulin accumulation around each nuclei. Total time: 27.3 hours; time interval: 10 min.

**Movie S3B.** Same movie as Movie S3A to show budding from PGCC nucleus in the absence of GFP-labeled tubulin.

**Movie S4.** Apoptosis of a mononucleated PGCC. The multinucleated PGCC formed rosette-like structure with centrally located centrosome-like dense microtubulin spot in the center and continue endocycle within cytoplasm before apotosis. Nucleus: H2B-RFP, red; spindle:α-tubulin-GFP, green. Total time: 27.8 hours; time interval: 10 min.

**Movie S5A.** Budding of a chromatin bulge from a mononucleated PGCC nucleus in the absence of mitosis and travel inside cytoplasm toward the surface and subsequently matured into a functional cell capable ot mitosis. The movie showed cell and nuclear size as an indication of endocycle. Total time: 10.5 hours; time interval: 10 min.

**Movie S5B.** Same movie as S3A to show RFP-histone 2B-budding of mononucleated PGCC in the absence of GFP-labeled histone.

**Movie S6A.** Budding of multiple multinucleated daughter cells from one nucleus (right) of a binucleated PGCC. One of the daughter cells acquired spindle morphology. Total time: 17 hours; time interval: 10 min.

**Movie S6B.** Same movie as S4A showing budding from the PGCC nucleus in the absence of GFP-labeled tubulin.

**Movie S7.** Early budding of daughter nuclei from a multinucleated PGCC followed by cytofission (splitting or cleavage). The budded daughter nuclei traveled within the cytoplasm toward the cell surface, and then mother multinucleated PGCC underwent asymmetric cytofission to generate two daughter cells with unequal numbers of nuclei. Total time: 33 hours; time interval: 10 min.

**Movies S8-S13.** Tracking of PGCC growth and division using FUCCI-labeled Hey cells. Movie S10, no paclitaxel treatment; Movies S11-S16, paclitaxel-treated cells.

**Movie S8.** Mitosis without paclitaxel treatment. Cells in the G1 phase are shown in red; cells in the G1 to S transition are shown in yellow; cells in the S, G2, and early M phases are shown in green; and cells in the late M phase to early G1 phase are colorless. Total time: 22 hours; time interval: 15 min.

**Movie S9.** Two endoreplication cell cycles generated a mononuclated PGCC. Total time: 77.5 hours; time interval, 30 min.

**Movie S10.** A mononucleated PGCC underwent endomitosis with budding to generate a multinucleated PGCC that continued endocycle. Total time: 145 hours; time interval: 30 min.

**Movie S11.** A multinucleated PGCC underwent endomitosis to generate multinucleated PGCCs that can further grow via endocycle. Note that the individual nuclei within the PGCC were different from each other in phases of cell cycle, suggesting that DNA replication of different nuclei within same PGCC is asynchronous. Total time: 95 hours; time interval: 30 min.

**Movie S12.** Two multinucleated PGCCs underwent endocycle. Total time: 52.5 hours; time interval: 15 min.

**Movie S13.** Resumed mitosis in PGCC-derived daughter cell. Total time: 40 hours; time interval time: 30 min.
